# Supplementary figures and images for: Isotretinoin promotes elimination of translation-competent HIV latent reservoirs in CD4T cells
Source: PLoS Pathog. 2024 Oct 14;20(10):e1012601. doi: 10.1371/journal.ppat.1012601 (PMC11501018; doi:10.1371/journal.ppat.1012601)

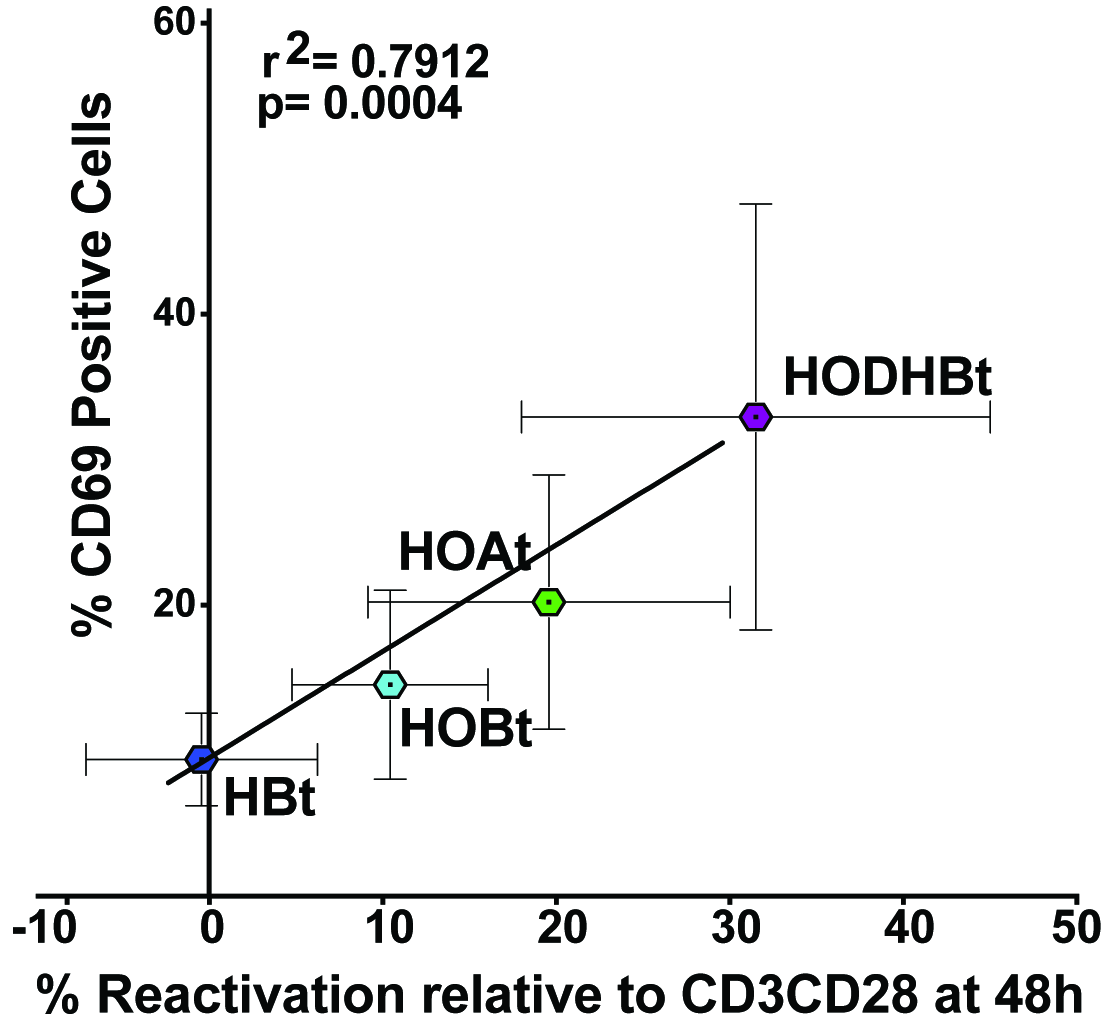

Supplement: S1 Fig — Levels of CD69 positive cells 24 hr after treatment of cultured TCM in 5 different donors were correlated with levels of HIV-1 reactivation at 48 hr. Error bars indicate SD. Partial data from Bosque et al, Cell Reports, 2017. (TIF) [file ppat.1012601.s001.tif]

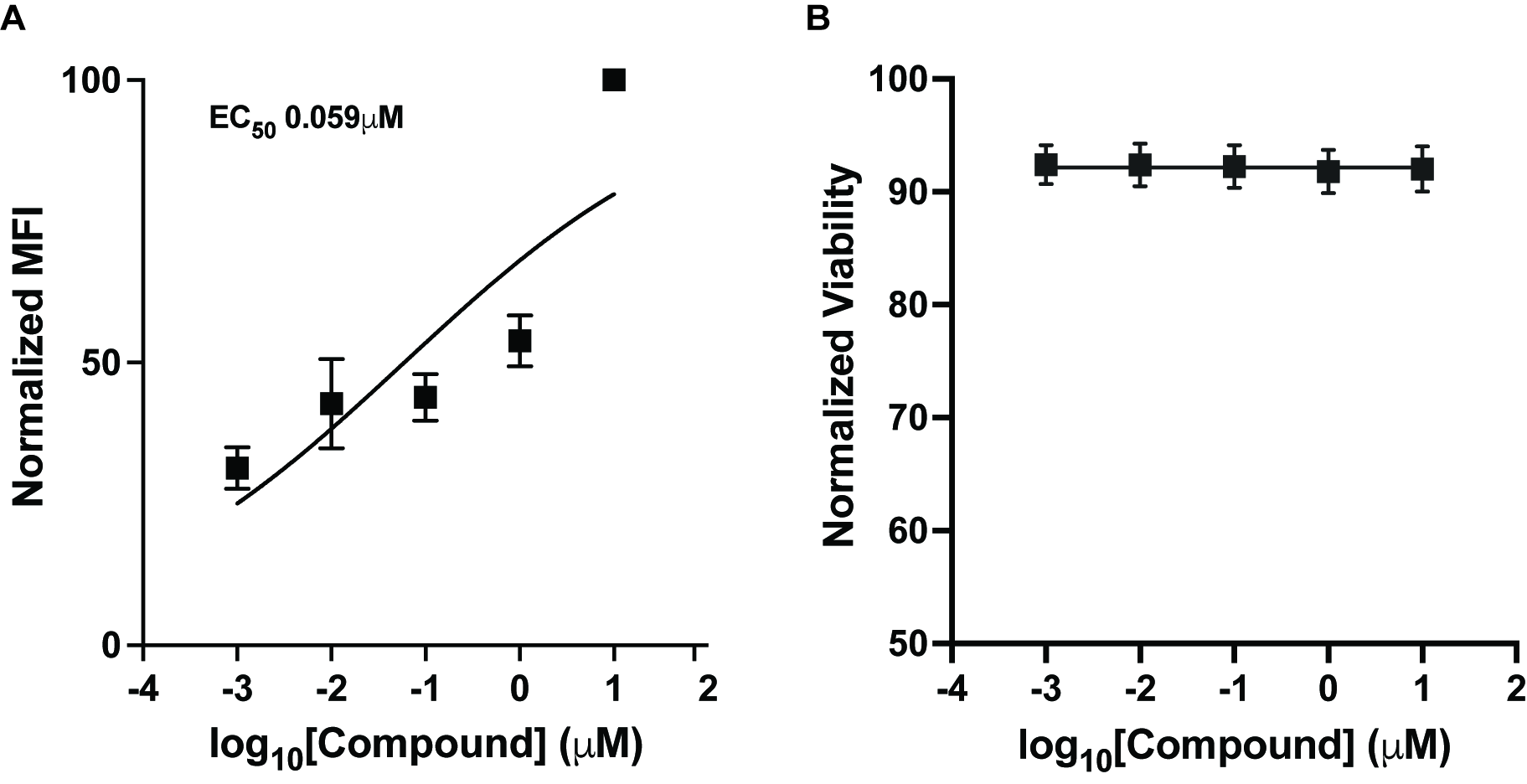

Supplement: S2 Fig — A: CD69 normalized MFI of matched cultured uninfected TCM (n = 4) were measured by flow cytometry after treatment with a dose response of Isotretinoin in the presence of 30 IU/mL IL-2 for 72 hours. B: Normalized viability of Isotretinoin dose response from (A). Error bars indicate SEM. (TIF) [file ppat.1012601.s002.tif]

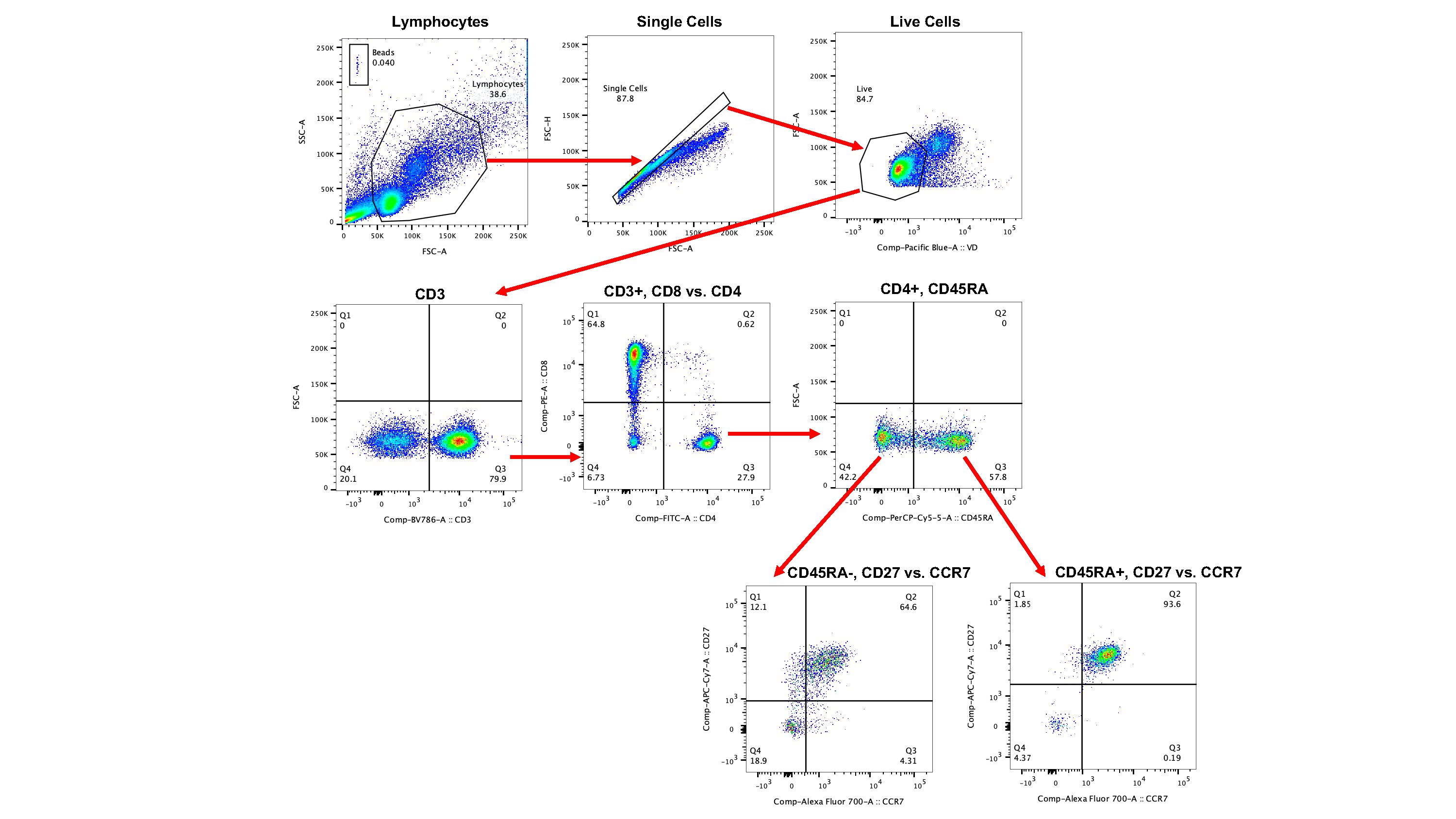

Supplement: S3 Fig — (TIFF) [file ppat.1012601.s003.tiff]

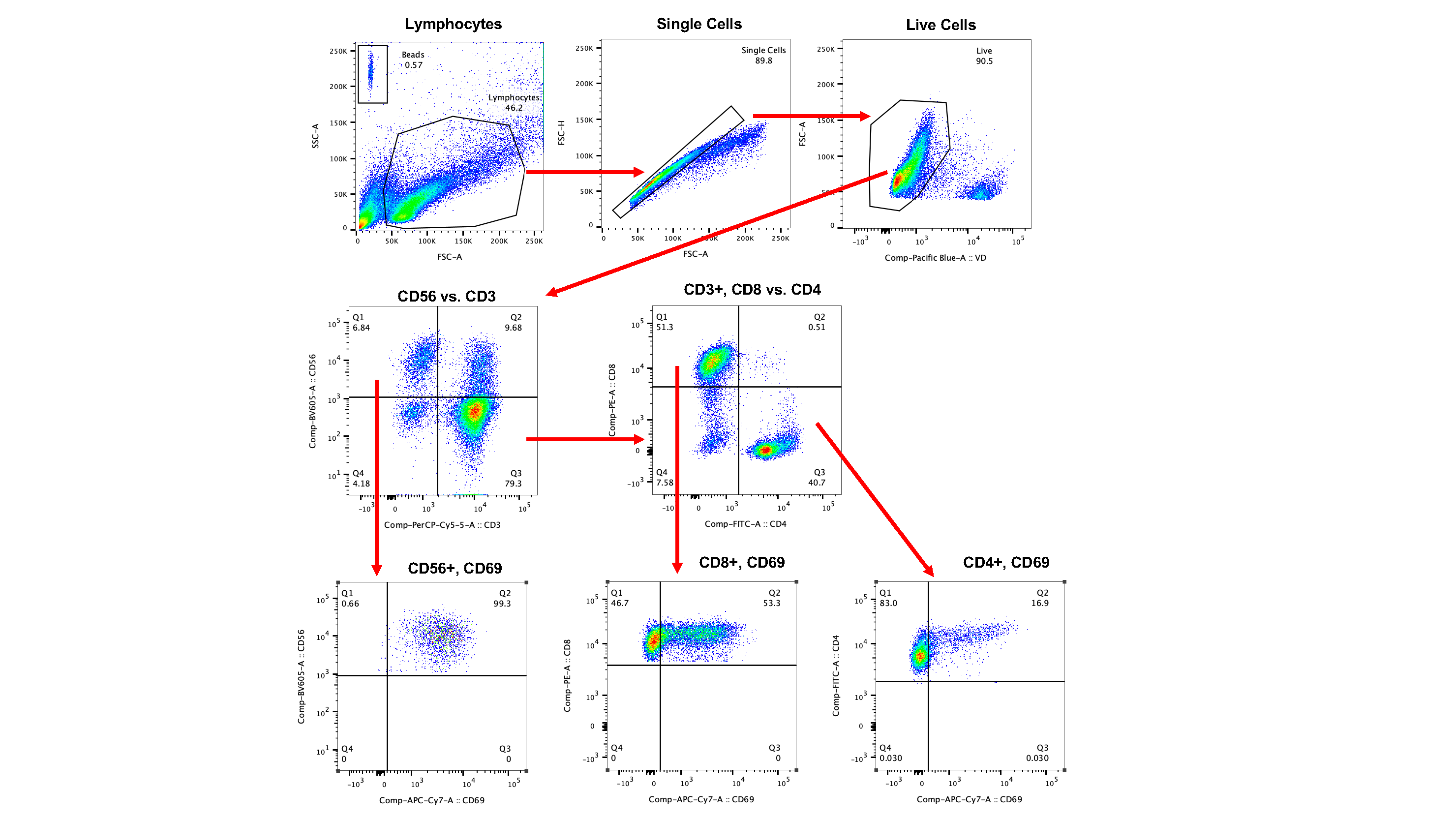

Supplement: S4 Fig — (TIFF) [file ppat.1012601.s004.tiff]

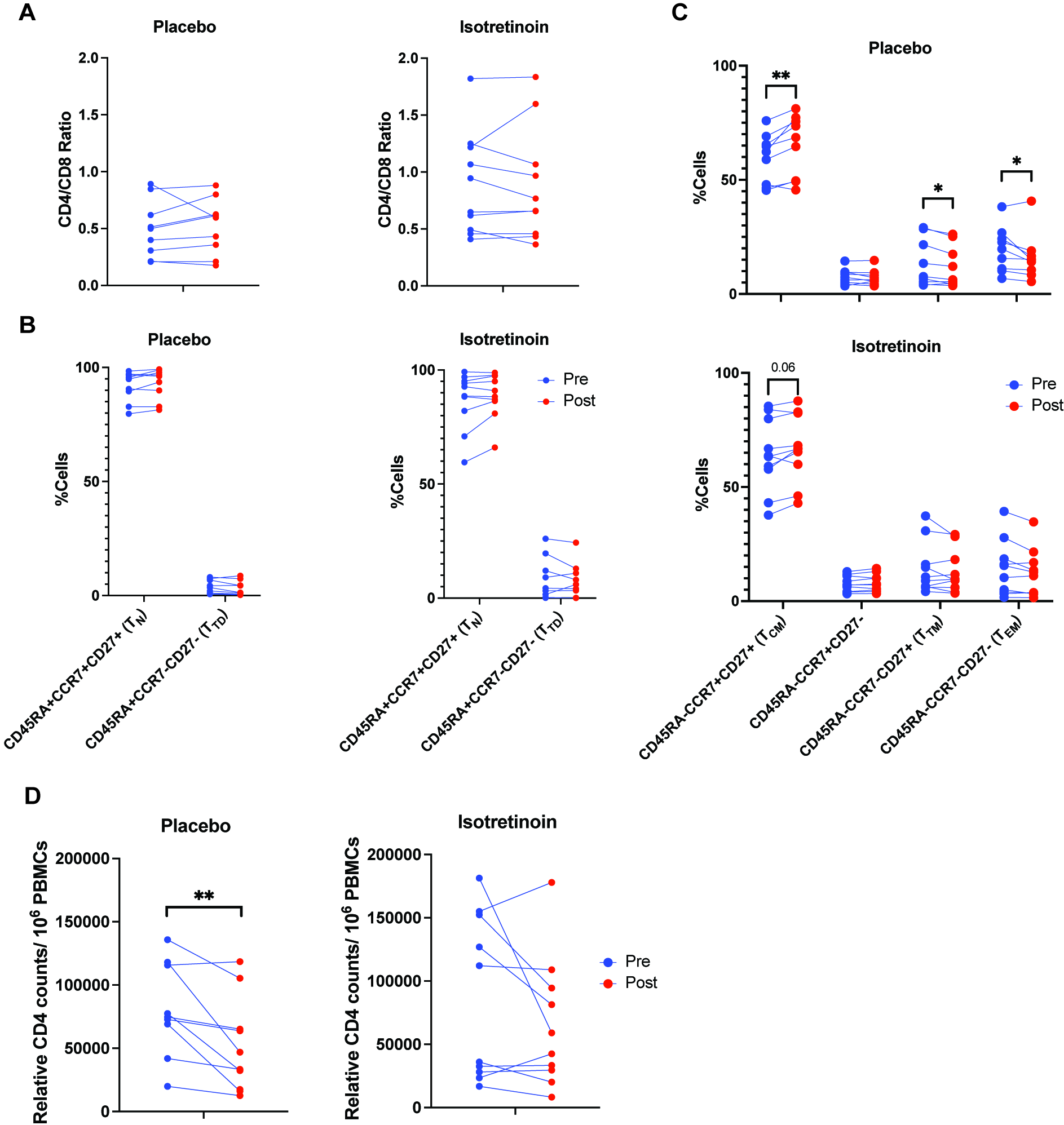

Supplement: S5 Fig — CD4/CD8 ratio (A), CD45RA+ subset phenotyping (B) and CD45RA- subset phenotyping (C) of PBMCs for each participant/ time point prior to reactivation stimulation. D: CD4 counts normalized to 106 PBMCs plated for each participant/ time point. Wilcoxon matched-pairs signed rank test was used to calculate p values (*p < 0.05; **p < 0.01). (TIF) [file ppat.1012601.s005.tif]

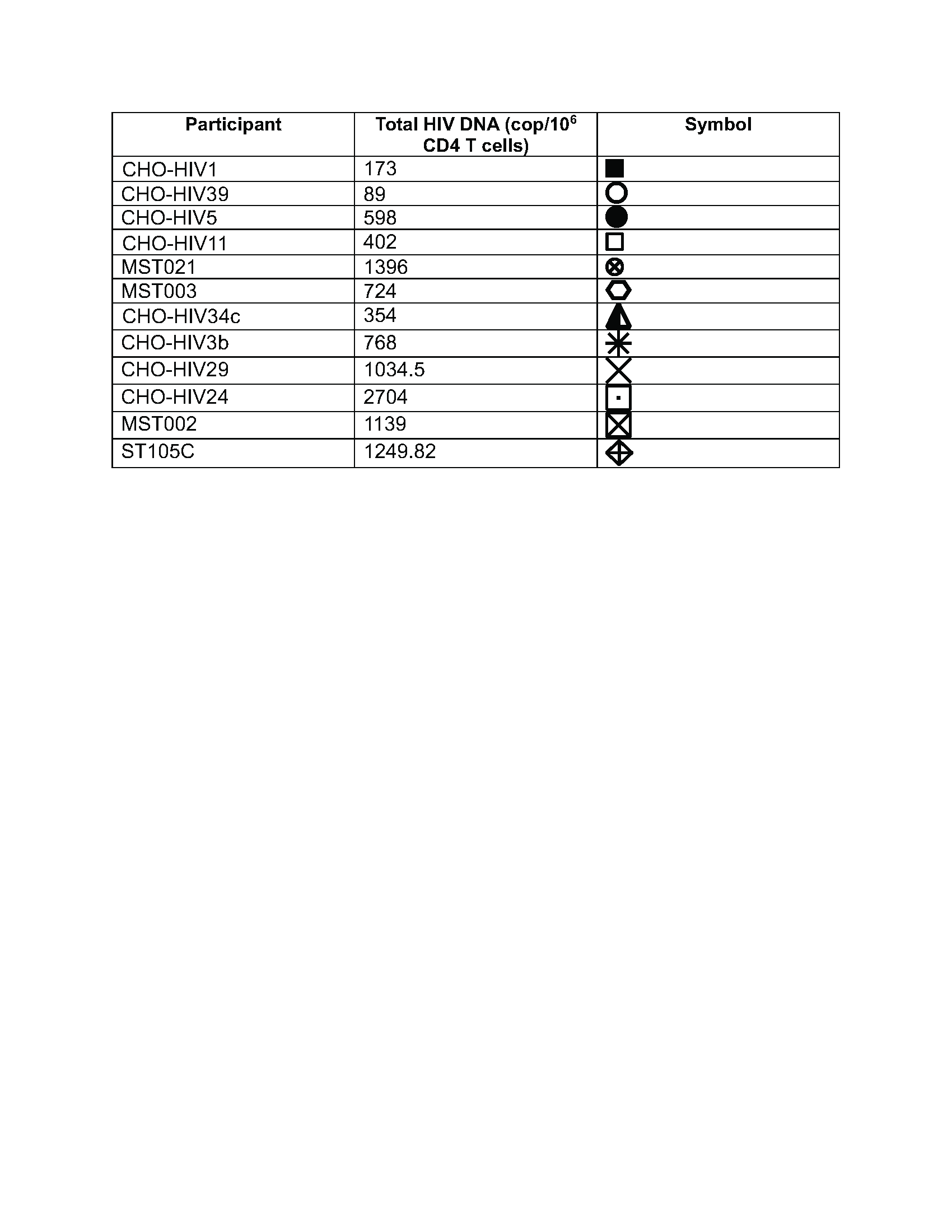

Supplement: S6 Fig — (TIFF) [file ppat.1012601.s006.tiff]

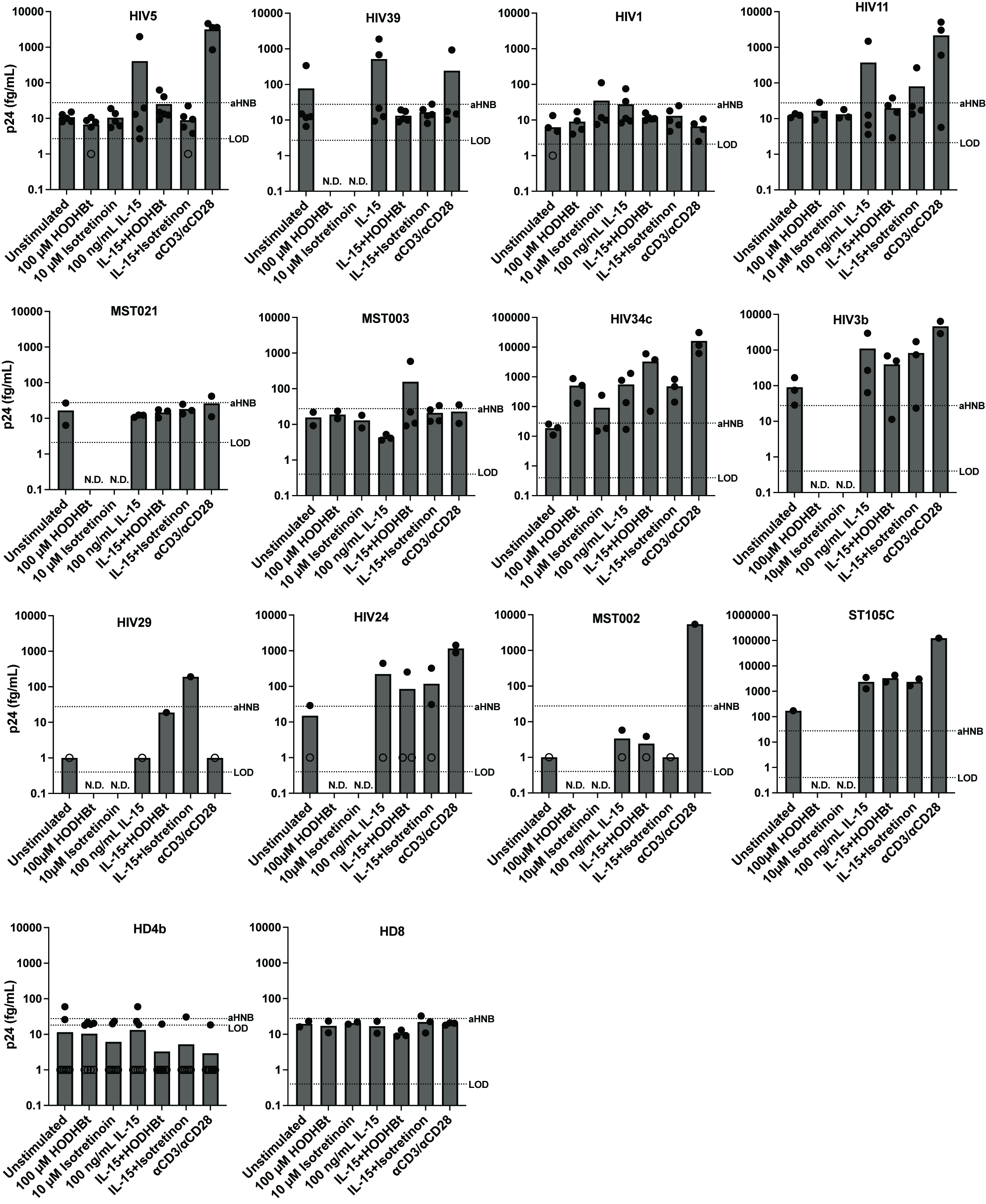

Supplement: S7 Fig — Individual p24 results in supernatants for 12 ART- suppressed PWH (HIV/MST) and 2 HIV negative participants (HD). Open circles denote wells with non-quantifiable p24 measurements as described in the methods. LODs listed for respective analysis runs, output by Quanterix software. Average HIV-negative baseline (aHNB) calculated as described in methods. N.D not determined. (TIF) [file ppat.1012601.s007.tif]

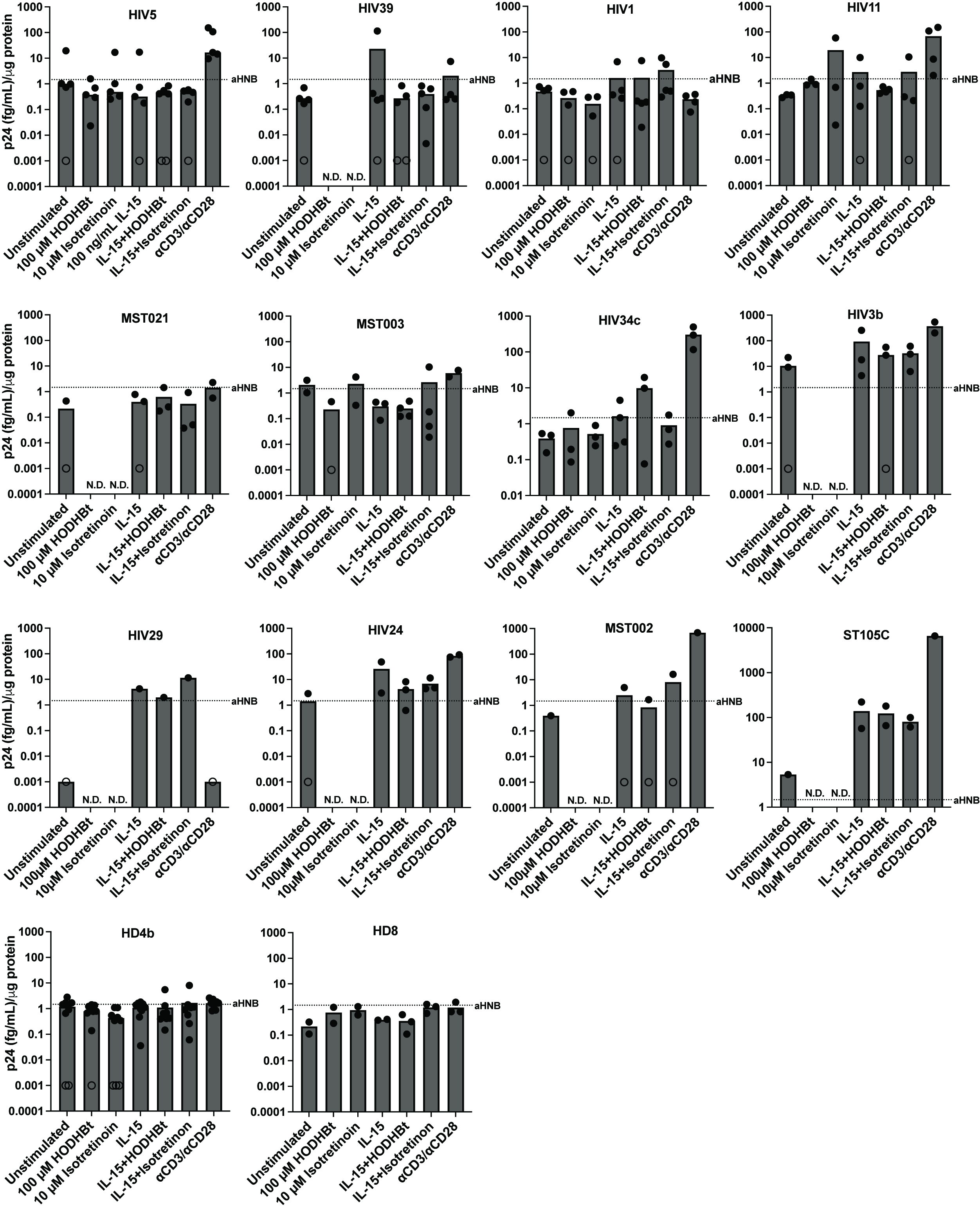

Supplement: S8 Fig — Individual p24 results in cell extracts for 12 ART- suppressed PWH (HIV/MST) and 2 HIV negative participants (HD). Open circles denote wells with non-quantifiable p24 values but where protein was measure via BCA as described in methods. Average HIV-negative baseline (aHNB) calculated as described in methods. N.D not determined. (TIF) [file ppat.1012601.s008.tif]

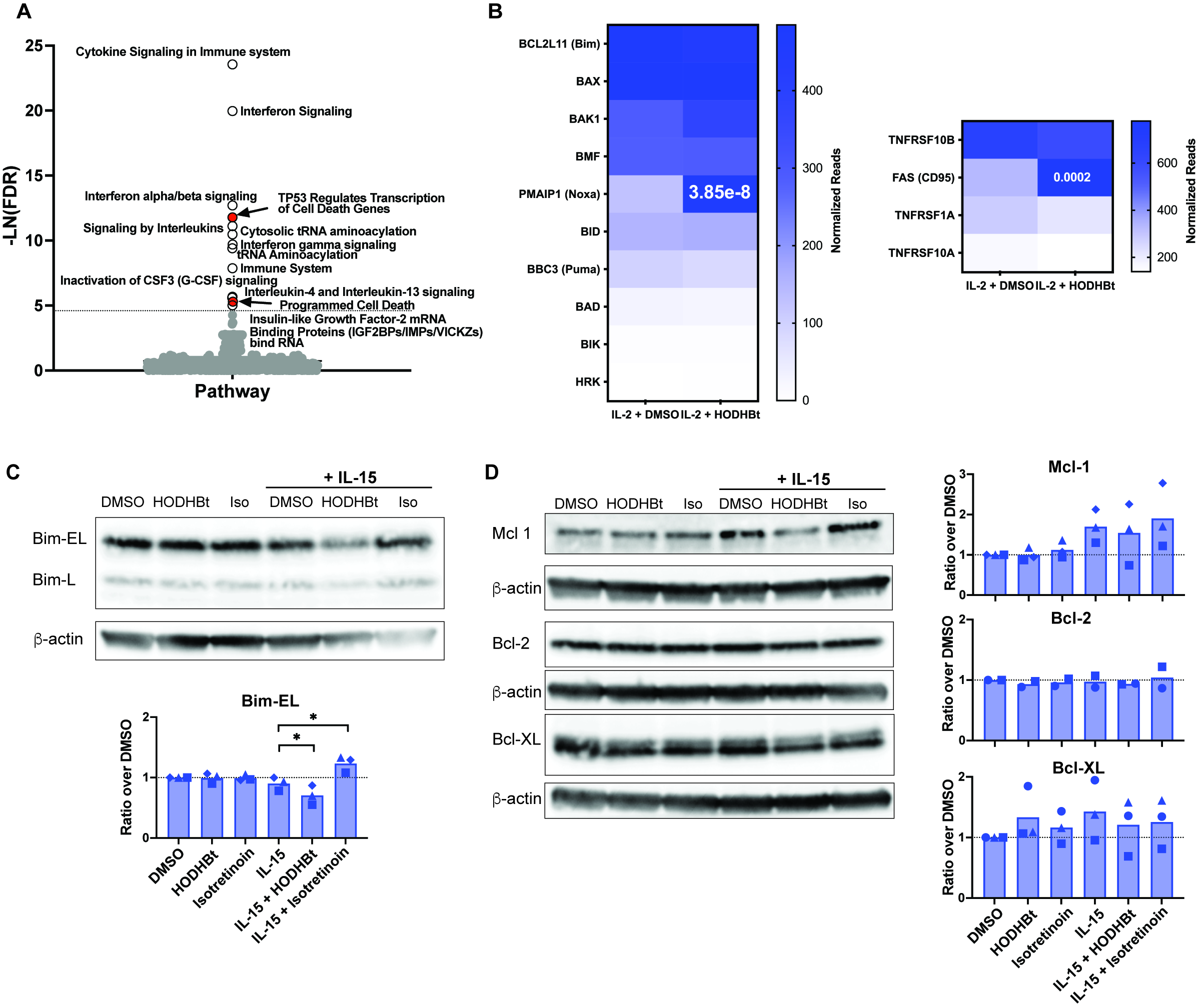

Supplement: S9 Fig — A: Reactome analysis of pathways modulated after HODHBt treatment. B: HODHBt treatment upregulates Noxa and CD95 in CD4 T cells. C and D: Levels of anti- and pro-apoptotic proteins were measured in cultured uninfected TCM treated with 100μM HODHBt or 10μM Isotretinoin alone, plus 100 ng/mL IL-15 for 24 hours (n = 2–3). Paired t-test was used to calculate p values (*p < 0.05). (TIF) [file ppat.1012601.s009.tif]

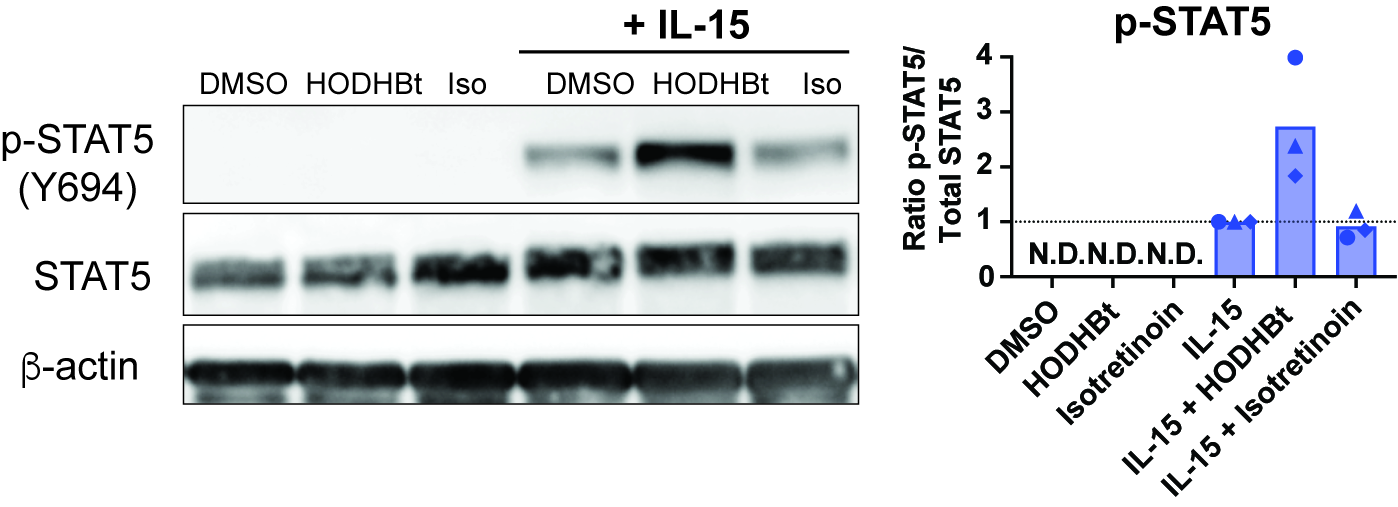

Supplement: S10 Fig — Levels of phosphorylated STAT5 and total STAT5 were measured in cultured uninfected TCM after treatment with 100μM HODHBt, 10μM Isotretinoin alone or plus 100ng/mL IL-15 for 24 hours (n = 3). (TIF) [file ppat.1012601.s010.tif]
