# Supplementary material for: Isotretinoin promotes elimination of translation-competent HIV latent reservoirs in CD4T cells
Source: PLoS Pathog. 2024 Oct 14;20(10):e1012601. doi: 10.1371/journal.ppat.1012601 (PMC11501018; doi:10.1371/journal.ppat.1012601)
Supplement: Attachment — Submitted filename: Response to Reviewers.docx [file ppat.1012601.s012.docx]

**Reviewer comments:**

We would like to thank the reviewers for taking the time to review our manuscript and provide insightful comments. We have addressed all of their concerns and we believe this has increased the quality of our manuscript. Below, please find a detailed response to the comments.

**Reviewer #2:**

Line 40-42: There have been cases of sterilizing cure after SCT from a donor who was not CCR5d32, and also from an individual who received no SCT.

We have now included the latest case of HIV eradication after bone marrow transplant. We have only included these cases as the eradication was achieved after a procedure. We are aware of other cases on where eradication was achieved naturally but the causes are unclear. We will appreciate the inclusion of any other published case that the reviewer may indicate.

Also, based on the new NIAID guidelines on HIV language to avoid stigmatization, we have replaced the term sterilizing cure for HIV eradication

83-85: Here, they could cite Kadiyala GN et al, AIDS, 2024.

We apologize for missing this important study. This citation has been added as suggested.

104-105: This sentence is somewhat confusing. You make it sound as if HODHBt stimulates the gc-cytokine pathway, but later on, you say that it does little unless it is combined with a gc-cytokine.

We thank the reviewer for the comment, and this has been clarified in the text.

113-115: Is it fair to compare gene expression changes in primary cells and cell lines?

We agree with the reviewer that the comparison of transcriptional changes observed in primary cells and cell lines may not be the most appropriate. Unfortunately, to our knowledge, there is not a database of transcriptional changes mediated by different drugs in primary cells. The strength of the CMap is that is comprised of multiple different cell lines in order to negate potential bias. Further validation is required to validate potential hits. In this study, we only evaluated 5 of the potential hits identified and only 1 was confirmed.

120-126: While the authors have previously shown an association between CD69 expression and levels of both pSTAT5 and reactivation, there might not be any causal connection. Thus, the relevance of CD69 here is somewhat unclear. Moreover, CD69 has been suggested to be both an early marker of T cell activation and a marker of tissue retention.

We agree with the reviewer about the dual role of the c-type lectin CD69 as an early activation marker but also a marker tissue retention marker. We chose to use this marker to begin screening the different FDA approved compounds for two reasons: 1) our previous findings showed that HODHBt enhances CD69 expression in the presence of a γc-cytokine (Bosque et al, Cell Reports 2017), 2) there are available flow cytometry antibodies for easy screening of levels of CD69 in the surface of primary CD4 T cells. We used this as our first step in validating the CMap hits, which as the reviewer previously pointed out, were generated from cell line data.

Fig S2: What is the “normalized viability?”

The normalized viability is the viability of the Isotretinoin concentrations relative to the DMSO control. This has been clarified in the figure legend.

126-128: The EC50 was calculated based on induction of CD69, which is not the most relevant marker. Did you measure the EC50 for any changes in HIV expression or killing of infected cells?

The EC50 for Isotretinoin was only calculated based on CD69 induction in this study. Because of the limitation of working with primary cells, we were unable to calculate the EC50 for each of the different measurements in this study.

128-130: The concentration of isotretinoin – 10uM – seems high. Can this concentration be achieved in vivo?

Based on what we were able to find in the literature, the serum concentration Isotretinoin has been shown to reach when administered orally to treat severe acne is at least 2μM (Almond-Roesler et al, Dermatology 1998, PMID: 9557257). Isotretinoin is one of multiple retinoids under investigation and in clinical trials. Our ongoing studies are evaluating other potential retinoids with higher activity

142-143 and Fig 1E: When you say “uninfected,” do you mean cells not exposed to virus? By latently infected, do you mean the virus-exposed cells, which would consist of a mixture of uninfected and latently infected?

We apologize for the confusion. Yes, the uninfected cells have never been exposed to virus, but all steps are carried out in parallel to those that were infected. The “latently infected” cells have been exposed to virus, and then sorted for CD4+ cells prior to reactivation. This population contains cells that are either completely uninfected or cells that were latently infected (not expressing p24 and downregulating CD4) prior to reactivation.

Fig 1E: To assess the effect of isotretinoin on viability, it looks like the isotretinoin+IL-15 was compared between the uninfected and latent cells. It seems like it would be better to compare the same cell type that was or was not exposed to isotretinoin (i.e.: DMSO+IL-15 vs. isotretinoin+IL-15).

We thank the reviewer for the comment. We performed these comparisons (shown below) and there was no significant difference in the viability of the uninfected cells between IL-15 and either the HODHBt + IL-15 or Isotretinoin + IL-15 conditions. In the latently infected cells we observed a significant increase in viability after addition of HODHBt to IL-15, but no effect on viability after addition of Isotretinoin to IL-15 (Wilcoxon matched pairs signed rank test used).

162-165: Why did placebo increase Tcm and decrease Ttm and Tem?

We thank the reviewer for the comment and had the same question. Given that there should be no biological effect of the placebo control group, we hypothesize that the observed increase in the T_CM_ population and subsequent decrease in the transitional and effector memory populations might just be due to the inherent turnover of T cells. But we cannot speculate any further based on the limited amount of samples provided from the clinical trial.

171-173, Fig 2A: Did you look at other markers of activation, such as HLA-DR+CD38? Rather than compare the pre- and post-placebo, it seems like it would be better to compare not just the pre- and post-isotretinoin (as you did), but also to compare the post-placebo and post-isotretinoin.

We thank the reviewer for the question. Unfortunately, we did not look at additional markers of activation, only CD69. Additionally, we did not observe any significant differences in activation of CD4s, CD8s, or NK cells when comparing post-treatment conditions (shown below).

174-177: The way this sentence is written, it is not clear that both of these observations came from the placebo arm. Perhaps you should say, “In the placebo arm, we saw a significant increase in both…” Why do you see these changes after placebo?

We thank the reviewer for the comment. This sentence has been clarified in the updated manuscript. Like the point mentioned above, placebo should have no biological effect on activation. Therefore, we can only hypothesize that the changes observed in the placebo group are due to the inherent donor-specific intracellular fluctuations in cell activation. We don’t have any further information regarding the participants in the study or other potential clinical complications that may be affecting T cell activation.

177-180: If not already done, it would be best to compare post-isotretinoin to post-placebo.

We thank the reviewer for the comment and these comparisons are shown above.

Fig 2B: Here, it would be helpful to show the median values. In the cells from pre- and post-placebo, was there any difference between the unstimulated and IL-15 conditions? In the post-placebo, was there any difference between the unstimulated and anti-CD3/CD28? In the pre-isotretinoin, was there a difference between the unstimulated and IL-15? I would expect differences.

We have now included the median values.

We did all those comparisons and only those that reach statistical significance were shown (Wilcoxon matched-pairs signed rank test used). A potential explanation is that the number of participants in each arm is limited and there is variability to the response to both IL-15 and CD3CD28.

Fig 2C: In addition to comparing the pre- and post-, you should also compare the post-placebo to post-isotretinoin.

We thank the reviewer for the suggestion. Comparison of the post-placebo for the IL-15-stimulated and αCD3/28-stimulated conditions showed no statistically significant differences in the % increase of p24 over unstimulated (graphs shown below: Wilcoxon matched pairs signed-rank test).

198-199: Did you measure viability directly, in addition to measuring LDH in supernatant?

We thank the reviewer for the question. Because our objective was to also measure p24 in the cell lysates, we could not detect viability directly as the cells are lysed with a detergent based buffer. Viability was then only measured by LDH in the supernatant.

207-209 and Fig 3: The results from Fig 3 seem to contradict those from Fig 2. Both experiments were performed ex vivo, and both were analyzed at the same time point. In Fig 2, the data suggest that cells from isotretinoin-treated PWH show an increase in p24 after ex vivo treatment with IL-15. However, in Fig 3A-B, ex vivo treatment with isotretinoin+IL-15 resulted in significantly less p24 in the supernatant than IL-15 alone, and there was a similar trend in the cell lysates. I understand the authors’ explanation of Fig 3 - that isotretinoin+IL-15 is leading to killing of p24+ cells - but it could also be that ex vivo treatment with isotretinoin actually reduces the effect of IL-15 on increasing p24 expression, or that both are true. Conversely, if ex vivo treatment with isotretinoin increases the killing of p24+ cells after IL-15 exposure (Fig 3), why does in vivo treatment with isotretinoin not lead to a similar decrease in p24 after ex vivo treatment with IL-15 in Fig 2? Could it be that the effect of isotretinoin to augment death of p24+ cells is not observed in vivo? To test your theory, can you do a time course experiment where you take cells from the isotretinoin trial, treat ex vivo with IL-15, and show that the p24 first goes up and then comes down over time? Alternatively, can you measure HIV DNA over time or compare to negative control to show killing of infected cells?

We thank the reviewer for the comments and questions. We agree that the results in Figure 2 and 3 are somewhat contradictory and are open to many interpretations like the reviewer suggests.

First, based on primary cell model of latency, we do not believe that isotretinoin negates the effects of IL-15 (Fig 1C). As we have shown, isotretinoin enhances IL-15 latency reversal in this model. Second, it is important to point that while both experiments were treated ex vivo, the data in Figure 2 is from cells from participants that were treated with Isotretinoin in vivo, and then exposed with IL-15 ex vivo. The participants in the clinical trial received Iso for 16 weeks before the post-treatment time point was collected. For our experiments, cells are thoroughly washed after thawing and the levels of isotretinoin left in the cells is unknown but most likely gone. Contrastingly, the cells used in Figure 3 were treated ex vivo with both isotretinoin and IL-15 at the exact same time, so both compounds were able to exert their direct biological effects in tandem. Ultimately, we are suggesting that the timing of treatment with both Isotretinoin and IL-15 is important in determining which outcome in terms of p24 expression is observed, and to answer this question a new clinical trial is needed that measures the effects of treating with IL-15 and Isotretinoin simultaneously. Regarding the experiments proposed by the reviewer, unfortunately we do not have access to any additional cells from the clinical trial to test this hypothesis.

220-222: As above, another explanation is that isotretinoin actually reduced the ability of IL-15 to induce p24 expression ex vivo.

This could be a possible explanation for the observation as well but based on our data in the primary cell model, we do not believe that is the case.

231-233 and Fig 4A: To clarify, is the data shown from day 24-26, after activation with anti-CD3/CD28? Why use the paired T test instead of the Wilcoxon?

Yes, the data shown is after the second reactivation with only the αCD3/CD28 beads. We used the paired T test due to the small sample size.

242-244: Based on Fig 4, isotretinoin did not seem to increase expression of Noxa unless it was combined with IL-15.

We thank the reviewer for the comment. As with HODHBt, our data does suggest that Isotretinoin requires concomitant IL-15 to enhance Noxa and exert apoptotic effects.

250-253 and Fig 4D: Rather than saying “HIV-negative” and “HIV-positive,” I would say p24- and p24+. The term HIV-negative suggests the cells are not infected, but some may be infected and not expressing p24.

We thank the reviewer for the comment, and this has been clarified in the text accordingly.

255-258: By “HIV-negative,” I’m guessing you mean p24-?

We thank the reviewer for the question. Yes, we are referring to p24- here and this has been adjusted in the text.

257-258: You should say that isotretinoin only sensitizes HIV Gag-expressing cells to cell death.

We thank the reviewer for the comment, and this has been changed in the manuscript accordingly.

300: Instead of HIV-infected, I would say HIV p24-expressing.

We thank the reviewer for the comment, and this has been updated in the text.

328-330: Did prior publications from the isotretinoin trial show any data about levels of total HIV DNA, intact/defective HIV DNA, or IUPM from QVOA? If so, it would be helpful to remind the readers of these results in the Intro.

To our knowledge, the results of the clinical trial are now under review in JCI. There were measurements of HIV DNA but the results are unknown to us.

440: Do the activating beads fall off before 96h? If not, why did you do the magnetic separation? If the beads did not fall off, I would imagine that some cells (including many of the activated ones) would remain stuck to the magnet, while other cells (including many that did not get activated) would be removed in the supernatant. Which cells were lysed and analyzed? Was it the non-magnetic ones? Why not measure p24 in all of the cells together?

We thank the reviewer for the question. The magnetic separation was performed to separate the beads from all cells, so they didn’t interfere with downstream analysis. To our knowledge no significant proportion of cells remain left behind with the beads after separation because the wells are resuspended thoroughly before placing on the magnet. Our previous studies suggest that after prolonged activation, the majority of the cells are not attached to beads but there is always the possibility that some cells are left behind. After the beads are removed, cells and supernatant are also separated by centrifugation so no cells are analyzed in the supernatants.

441: Which cell pellet – the magnet bound cells, or the non-magnetic ones?

We thank the reviewer for the question. See above explanation

452-455: This sentence is confusing.

We thank the reviewer for the comment. Our intention here was to set a threshold using the p24 values from HIV-negative participants which should theoretically be 0. Due to the sensitivity of the assay being in the fg/mL range, we decided to add the value of 2 standard deviations to the baseline threshold to limit the number of potential false positive hits. This means that any value above the HIV-negative baseline we have 95% confidence that is a true positive and not an artifact of the extreme sensitivity of the assay.
